# The Role of Unfolded Protein Response and Mitogen-Activated Protein Kinase Signaling in Neurodegenerative Diseases with Special Focus on Prion Diseases

**DOI:** 10.3389/fnagi.2017.00120

**Published:** 2017-05-01

**Authors:** Syed Zahid Ali Shah, Deming Zhao, Tariq Hussain, Lifeng Yang

**Affiliations:** National Animal Transmissible Spongiform Encephalopathy Laboratory and Key Laboratory of Animal Epidemiology and Zoonosis of Ministry of Agriculture, College of Veterinary Medicine and State Key Laboratory of Agrobiotechnology, China Agricultural UniversityBeijing, China

**Keywords:** prion diseases, prion protein scrapie (PrP^Sc^), endoplasmic reticulum (ER), unfolded protein response (UPR), mitogen-activated protein kinases (MAPKs), neurodegenerative diseases

## Abstract

Prion diseases are neurodegenerative pathologies characterized by the accumulation of a protease-resistant form of the cellular prion protein named prion protein scrapie (PrP^Sc^) in the brain. PrP^Sc^ accumulation in the endoplasmic reticulum (ER) result in a dysregulated calcium (Ca^2+^) homeostasis and subsequent initiation of unfolded protein response (UPR) leading to neuronal dysfunction and apoptosis. The molecular mechanisms for the transition between adaptation to ER stress and ER stress-induced apoptosis are still unclear. Mitogen-activated protein kinases (MAPKs) are serine/threonine protein kinases that rule the signaling of many extracellular stimuli from plasma membrane to the nucleus. However the identification of numerous points of cross talk between the UPR and MAPK signaling pathways may contribute to our understanding of the consequences of ER stress in prion diseases. Indeed the MAPK signaling network is known to regulate cell cycle progression and cell survival or death responses following a variety of stresses including misfolded protein response stress. In this article, we review the UPR signaling in prion diseases and discuss the triad of MAPK signaling pathways. We also describe the role played by MAPK signaling cascades in Alzheimer’s (AD) and Parkinson’s disease (PD). We will also overview the mechanisms of cell death and the role of MAPK signaling in prion disease progression and highlight potential avenues for therapeutic intervention.

## Introduction

The transmissible spongiform encephalopathies (TSEs) are a group of fatal neurodegenerative disorders affecting both humans and animals. Major animals TSEs include bovine spongiform encephalopathies (BSE) in cattle, scrapie in sheep and goats and chronic wasting disease in elk and deer. Human TSEs include Creutzfeldt-Jakob disease (CJD), Gerstman-Sträussler-Scheinker syndrome (GSS), Fatal Familial Insomnia (FFI) and kuru (Nakagaki et al., 2013; Puig et al., 2016). Prion diseases have been the object of growing interest since the discovery of newly identified variant form of CJD (vCJD; Mukherjee et al., 2010). Prion diseases are characterized by the presence of an abnormal protease resistant misfolded isoform of the cellular prion protein termed as PrP^Sc^ (Prusiner, 1998). PrP^Sc^ is highly pathogenic and neurotoxic due to its β-sheet rich conformation as compared to predominantly α-sheet rich structure of PrP^c^ (Mukherjee and Soto, 2011; Song et al., 2016). TSEs are associated with the accumulation and aggregation of misfolded/unfolded disease-specific proteins in the brain leading to neuroinflammation and neurodegeneration. However the exact molecular mechanisms behind neuronal inflammation and apoptosis are still unclear and hence the development of an effective therapeutic strategy remains elusive (Moreno et al., 2013).

Endoplasmic reticulum (ER) is a multifunctional organelle responsible for folding and processing of secretory and transmembrane proteins. Balanced protein load and folding capacity are two important factors for correct protein folding and processing in the ER. Under pathological conditions this balance between folding load and capacity is disrupted and misfolded/unfolded proteins are accumulated that subsequently initiates ER stress (Beriault and Werstuck, 2013). ER stress activates an adaptive response known as the unfolded protein response (UPR) to correct misfolded/unfolded proteins and thus reduce ER stress. Long-term and severe ER stress dysregulates Ca^2+^ homeostasis and causes cytotoxic, synaptotoxic and behavioral changes (Mukherjee and Soto, 2011; Soto and Satani, 2011).

The exact molecular mechanisms leading to neurodegeneration in protein misfolding disorders including prion disease are still unclear. Compelling evidence suggests that one event that appears to be common to all neurodegenerative disorders is early neuroinflammation that contributes towards later neurodegeneration (Moreno et al., 2013). Microglia are activated by the oligomers and fibrils formed by amyloid proteins, including PrP^Sc^. Microglia are found clustered around amyloid plaques where they phagocytize and degrade plaques as an integral part of clearance mechanism (El-Shimy et al., 2015). Activated microglial cells control the release of proinflammatory mediators through activation of the MAPK signaling pathways (Kang et al., 2014). The basic structure of MAPKs is comprised of serine/threonine protein kinases which execute and promote a large number of cellular functions in various cell types. The mammalian cell is composed of three major subfamilies of MAPKs that transduces signaling within the cell or from cell surface towards inside of the cell. These three subfamilies are: the extracellular signal-regulated kinases 1 and 2 (ERK1/2), the c-JunNH_2_-terminal kinases (JNK) and the p38 kinases. The widely accepted perception that exists about these MAPKs is that JNK/SAPK (stress-activated protein kinase) and p38 MAPK help in promoting cell death in face of chronic stress stimuli, while ERK1/2 opposes cell death signaling cascades (Subramaniam and Unsicker, 2010).

Several research groups have focused on the role of MAPK pathways in all major neurodegenerative diseases such as Alzheimer’s disease (AD), Parkinson’s disease (PD) and prion diseases (Thellung et al., 2002; Ruano et al., 2006; Yang et al., 2014; Hwang et al., 2016). Amyloid beta (Aβ) chronically stimulate the ERK1/2 MAPK cascade via hippocampal alpha 7 nicotinic acetylcholine receptors (a7nAChRs). Uptake of RAGE- Aβ complex might be a promising therapeutic strategy for AD patients (Takuma et al., 2009). The molecular mechanisms regulating dopaminergic cell death in PD is not yet fully understood. Members of the MAPK family have been shown to be involved. In experimental PD models, the activation of JNK and p38 kinase precedes neuronal cell death in response to various stimuli (Newhouse et al., 2004). Hyperactivated p38 was found in postmortem brains of PD patients (Ferrer et al., 2001). Prion disease model employing SH-SY5Y cell line infected with PrP106–126 have shown apoptotic signaling mediated by p38 MAPK pathway (Thellung et al., 2002). It is have shown that clustering of PrP^c^ induces ERK1/2 pathway in GT1-7 neuronal cells (Monnet et al., 2004). Wang et al. (2005) have shown that p38 MAPK signaling cascade play a vital role in the induction of PrP^c^ in N2a cell line. More recently Puig et al. (2016) demonstrated that mutant prion protein is retained in secretory pathway through the induction of p38 MAPK pathway.

All major protein misfolding diseases, including AD, PD, Huntington’s disease (HD) and prion diseases have harmful effects on humans and animals due to lack of effective therapeutic strategies and presymptomatic diagnostic tools. Thus there is a need of novel therapeutic intervention strategies to control these diseases. Here in this review article, we will focus on the role of UPR and MAPK signaling cascades in neurodegenerative diseases with special focus on prion diseases.

## Unfolded Protein Response in Prion Diseases

**Figure 1:**
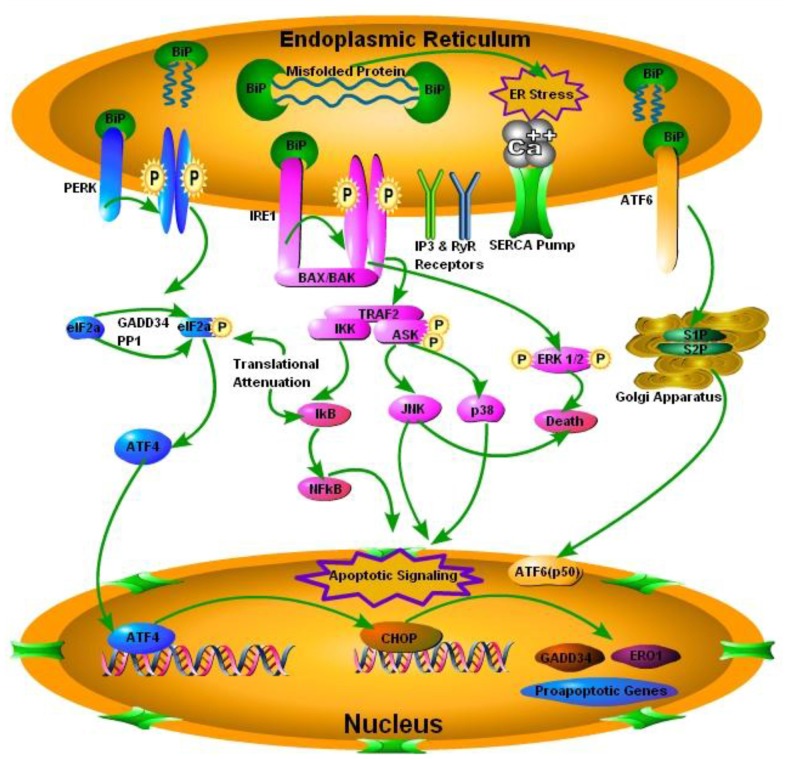
**Schematic presentation of the prion protein scrapie (PrP^Sc^) accumulation and subsequent endoplasmic reticulum (ER) stress mediated activation of the iinositol requiring enzyme 1 (IRE1), double stranded RNA-activated protein kinase (PKR)-like ER kinase (PERK) and activating transcription factor 6 (ATF6).** PERK pathway, through PERK phosphorylation and translation of initiation factor 2 (eIF2α) phosphorylation, inactivates eIF2α and attenuates translation via activating transcription factor 4 (ATF4), which ultimately results in activation of C/EBP homologous protein (CHOP) and pro-apototic, growth arrest and DNA damage-inducible protein 34 (GADD34) and ER oxido-reductin 1 (ER01) genes. IRE1 pathway activates apoptotic signaling via BCL2-associated X protein (BAX) and BCL2 antagonist/killer (BAK). TNF receptor associated factor 2 (TRAF2), results in the activation of IkB kinase (IKK) and apoptosis signal regulating kinase (ASK), IKK through inhibitory kappa B (IkB) activates nuclear factor of kappa B (NFkB) and ultimately apoptotic signaling occurs. On the other hand ASK phosphorylation results in the activation of c-Jun NH2-terminal protein kinase (JNK) and p38 pathway of mitogen activated protein kinase (MAPK) pathways. Phosphorylation of extracellular signal-regulated kinases 1 and 2 (ERK1/2) results in death via conjunction with JNK. Activating Transcription factor 6 (ATF6) pathway via site 1 and site 2 proteases (S1P and S2P) activates transcription factor p50 in nucleus.

Perturbations in the ER homeostasis contributes to the pathogenesis of several neurodegenerative diseases including prion diseases (Moreno et al., 2012). Accumulation of misfolded proteins in the ER activates the UPR to re-establish the balance between ER protein folding capacity and protein load resulting in cell survival. Chronic ER stress promotes cell death (Darling and Cook, 2014). The mechanisms of transition between cell survival and cell death during UPR are still unclear. The conversion of PrP^c^ to PrP^Sc^ is the hallmark of all TSEs (Mukherjee et al., 2010). While normal PrP^c^ is an integral part of the cell playing an important role in neuronal homeostasis, PrP^Sc^ is indeed considered harmful for cell survival and misfolded forms of PrP^Sc^ triggers the ER stress signaling (Figure 1; Soto and Satani, 2011). Yedidia et al. (2001) have shown that during transport through the ER, 10% of the native PrP^c^ is naturally converted into misfolded form. This shows how prone they could be to get misfolded (Yedidia et al., 2001). The importance of UPR in prolonged survival mechanisms was recently shown by Satani et al. (2016) where they demonstrated that mild activation of UPR through tunicamycin treatment prolonged the survival of prion infected mice. Apodaca et al. (2006) showed that UPR and ER associated degradation (ERAD) play an important role in the pathogenesis of prion diseases. Furthermore the absence of UPR and ERAD quality control impair cellular growth (Apodaca et al., 2006). There are three mechanistically distinct branches of the UPR. Each branch begins with a specialized stress sensor located at the ER membrane: inositol requiring enzyme 1 (IRE1), double stranded RNA-activated protein kinase (PKR)-like ER kinase (PERK), and activating transcription factor 6 (ATF6; Figure 1; Shah et al., 2015). The first branch of the UPR relies on the dimerization of IRE1 and its autophosphorylation to initiate a signaling cascade mediated through the transcription factor X-box binding protein 1 (XBP-1) to upregulate a subset of the UPR related genes involved in the protein folding processes and the ERAD (Mays and Soto, 2016). The second branch of the UPR is activated by the oligomerization of PERK protein that phosphorylates itself and the translation of initiation factor eIF2α. That in turn inactivating eIF2α and result in the translational shutdown. The third UPR pathway is initiated by the ATF6 following cleavage in the golgi apparatus, which increases the expression of the glucose regulated proteins (grp78) and the XBP-1 transcription factor (Hetz and Mollereau, 2014). To date most extensive work has been carried out on the IRE1 and PERK pathway in prion diseases and some minor work has been done on ATF6 pathway of the UPR.

The IRE1α/XBP-1 pathway is highly conserved amongst all eukaryotes. Orsi et al. (2006) have shown that under chemical-induced stress conditions in *in vitro* environment, the expression of a dominant negative form of the IRE1α or XBP-1 significantly increased PrP aggregation. While overexpression of an active mutant form of XBP-1 decreased the accumulation of misfolded PrP aggregates (Orsi et al., 2006). Similarly Hetz et al. (2008) have shown that prion infection of wild type mice led to the splicing of the XBP-1 mRNA and the activation of stress kinases mediated by the IRE1α pathway. To further investigate the role of IRE1α pathway in prion diseases, Hetz et al. (2008) designed an *in vivo* XBP-1 conditional knockout mice model. Interestingly, prion infection of XBP-1 knockout mice and wild type mice did not show any differences at the levels of prion replication and neuronal loss. Also there was no significant difference in upregulation of apoptosis markers or incubation periods (Hetz et al., 2008). These results suggest that the involvement of the UPR in prion disease is complex and possibly some compensatory pathways exist to deal with the damage. One may hypothesize that the activation of other UPR pathways may compensate for the XBP-1 deficiency, but there was no evidence that this occurred by the end-stage prion disease in XBP-1 knockout mice.

The main purpose of the PERK pathway signaling cascade is to relieve the ER stress by reducing the amount of proteins entering the ER (Shah et al., 2015). Moreno et al. (2012) have shown that PERK pathway took an active part during prion infection of the wild type mice and all the hippocampi of prion-infected wild type mice and those overexpressing PrP^c^ had activated PERK branch of the UPR. As PrP^Sc^ levels rise in PrP^c^ overexpressing mice infected with prions, there is global translational repression of the protein synthesis via phosphorylation of the eIF2α (eIF2α-P). The general decline in several synaptic proteins levels during infection was proposed to be a key trigger for neurodegeneration (Moreno et al., 2012). Similarly, DNA damage inducible protein 34 (GADD34) overexpressing mice model or chemical inhibition of the PERK by using PERK inhibitor GSK2606414 ameliorated neurodegeneration in prion-infected mice. On the other hand activation of the PERK pathway using salubrinal worsened prion associated neurotoxic events (Moreno et al., 2013; Halliday et al., 2015). However, since the PERK pathway can reduce the protein levels without altering the mRNA levels, ER stress induced translational repression of the PRNP remains a potential mechanism for the preclinical reduction in the PrP^c^ levels observed during prion diseases (Mays et al., 2015). Cohen et al. (2013) have shown that Snord3A is a consistent biomarker of prion disease in a mice model and Snord3A is directly correlated with the ATF6 levels in brain homogenates of prion infected mice. These studies highlight two critical points: (1) PERK activation leads to phosphorylation of eIF2α and subsequent inactivation of eIF2α occurs downstream to PrP^Sc^ replication in the prion diseased mice; and (2) reversing the translational repression of the synaptic proteins is a valid therapeutic strategy for prion disease.

## Triad of The MAPK Pathways

ER is a major calcium storing organelle within the cell that controls the ER stress through UPR signaling. Three branches of the UPR; IRE1α, PERK and ATF6 plays a central role in the initiation and regulation of UPR signaling (Shah et al., 2015). MAPK is a signal transduction pathway belonging to a large family of serine/threonine protein kinases. These kinases constitute major inflammatory signaling pathways from the cell surface to the nucleus. MAPK pathways are activated via phosphorylation of specific threonine and tyrosine residues mediated by MAPK kinases and on the other hand the dephosphorylation of MAPK phosphatases (MKPs) inactivates these pathways (Vasudevan et al., 2005; Koga et al., 2012; Taylor et al., 2013; Adachi et al., 2014). MKP1 regulates axon branching via deactivation of JNK signaling (Jeanneteau et al., 2010). Similarly MKP1 was found to be neuroprotective in midbrain dopaminergic neurons via inhibition of p38 MAPK. The role of MKPs in apoptotic signaling via dephosphorylation of ERK1/2 cannot be neglected (Kim et al., 2005). Recent reports have shown that MAPK signaling pathways have a major role in response to the ER stress but the complex nature of the UPR and MAPK pathways is still unclear (Figure 1). As mentioned earlier some MAPK pathways form a basic structural component of the UPR signaling cascade upon activation through any stress stimuli (such as p38 MAPK and c-Jun N-terminal kinase (JNK) pathways) and on other hand members of the MAPK pathway may promote adaptation, survival and resistance to the ER stress (such as ERK1/2; Figure 1). The MAPKs are proline-directed Ser/Thr protein kinases, distantly related to the cyclin-dependent kinases (CDKs). There are three well-characterized subfamilies of MAPKs: the extracellular signal-regulated kinases (ERK1/2), the c-Jun NH_2_-terminal kinases (JNK) and the p38 family of kinases (p38 MAPKs). They are activated through three-tier kinase signaling mechanism. As mentioned above, the MAPK subgroups are activated upon phosphorylation via the dual threonine/tyrosine motif in their activation loop: ERK1/2 with glutamine (Glu), proline (Pro) and glycine (Gly) embedded between threonine/tyrosine motif (Thr-Glu-Tyr), JNK (Thr-Pro-Tyr), and p38 (Thr-Gly-Tyr). There are a variety of selected MAP kinase kinases (MKKs) which phosphorylate these threonine/tyrosine motifs. The MKKs are in turn activated via the phosphorylation of a number of MAP kinase kinase kinases (MKKKs). The intricate mechanisms of biological outcomes of MAPK signaling are dependent on several factors including the sub-cellular localization of the MAPK signaling (determined by the amount of misfolded proteins and scaffolding proteins). In addition, the magnitude and duration of MAPK pathways activation and the effect of other signaling pathways such as UPR on MAPK pathways determines cell fate (Darling and Cook, 2014).

## A-The ERK1/2 Pathway

**Figure 2:**
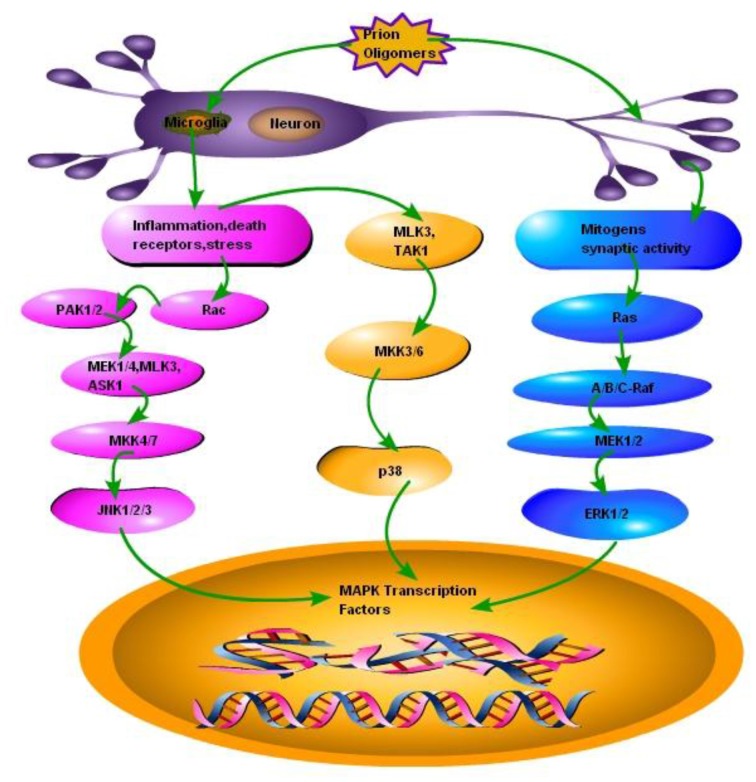
**Schematic presentation of a single neuron affected by the prion fibrils and oligomers.** Accumulation of misfolded PrP^Sc^ results in the activation of microglia and subsequently activating two types of responses: (1) Inflammation, stress and death receptors response; This response activates two pathways, one branch activates mixed-lineage protein kinase 3 (MLK3) and transforming growth factor activated kinase-1(TAK1), MLK3 and TAK1 activates mitogen-activated protein kinase kinase 3, 6 (MKK3, 6) MKK3, 6 activates the p38 branch of MAPK pathways, while the other branch of Inflammation, stress and death receptors response activates GTP binding protein (RAC), RAC activates P21 protein (Cdc42/Rac)-Activated Kinase 1 and 2 (PAK1, 2), PAK1, 2 activates mitogen activated protein kinase 1 and 4, MLK3 and apoptosis signal-regulating kinase 1 (ASK1), MEK1/4, MLK3 and ASK1 activates MKK4/7 to ultimately activate JNK branch of MAPK pathway. (2) Mitogens synaptic activity response; this branch activates small GTP binding proteins (Ras), Ras activates serine/theronine protein kinase (Raf A, B and C), Raf-A, B and C activates MEK1/2, which activates third branch of MAPK pathway ERK1/2.

ERK is a unique protein kinase which regulates several cellular functions ranging from cell survival to cell death signaling. It is established now that the duration and magnitude of ERK1/2 signaling determines the fate of the cell. The ERK Pathway is involved in the proliferation and differentiation of cell. It is also involved in neuronal plasticity, long-term potentiation and learning and memory (Obata and Noguchi, 2004). Some evidence suggests that ERK1/2 plays a major role in the promotion of cell death in a variety of neuronal systems which includes its involvement in neurodegenerative diseases (Uppington and Brown, 2008; Subramaniam and Unsicker, 2010). The ERK1/2 is activated in response to phosphorylation by the dual specificity MKKs, MAPK or mitogen activated ERK kinase1/2 (MEK1/2; Darling and Cook, 2014). Insulin or nerve growth factors (NGFs) were initially considered to act as stimulants for ERK1/2 activation (Subramaniam and Unsicker, 2010). Recent studies reported that ERK1/2 are virtually activated by almost all growth factors including those acting on several receptors such as tyrosine kinases, serine kinases, cytokine receptors, integrins and G-protein coupled receptors. The Ras activated serine/theronine protein kinase (RAF) activates MEK1/2 and this RAF-MEK1/2-ERK1/2 complex is a major target for the products of the Ras proto-oncogenes known as RAS GTPases (Figure 2). The outcome of signaling through ERK1/2 depends on the duration of activation and in part through the regulation and activation of the transcription factor activator protein (AP)-1. This leads to the DNA binding and transcriptional activation in the nucleus. The transient activation of ERK1/2 promotes the expression of c-Fos. While the expression of Fra-1, Fra-2, c-Jun and Jun B (AP-1 components) occurs after sustained ERK1/2 activation. These reactions ultimately promote phosphorylation of the C-terminal, trans-activation domain of c-Fos (Cook et al., 1999; Chalmers et al., 2007). As a consequence, sustained activation of ERK1/2 promotes cyclin-D1 expression and cell proliferation (Balmanno and Cook, 1999). In contrast, treatment with NGF promotes a very strong and sustained ERK1/2 activation that leads to apoptosis and terminal differentiation (Chipuk et al., 2010).

Apart from playing an active role in the control of main cellular functions of cell proliferation and differentiation, the ERK1/2 activation promotes cell survival in the face of a number of stress stimuli (misfolded proteins, Ca^2+^ dysregulation, nerve injury etc.) through the regulation of the BCL2 family members (Chipuk et al., 2010). For example the ERK1/2 dependent activation and transcription of the pro-survival proteins myeloid cell leukemia 1 (MCL-1), B-cell CLL/lymphoma 2 (BCL2) and B-cell lymphoma-extra large (BCL-XL). Most likely it occurs in response to the activation of the cAMP responsive element binding protein (CREB; Chipuk and Green, 2008). ERK1/2 dependent phosphorylation of MCL-1 leads to cellular stabilization. Furthermore ERK1/2 decreases the expression of several pro-apoptotic proteins such as Bcl-2-like protein 11 (BIM), p53 upregulated modulator of apoptosis (PUMA) and Bcl-2-modifying factor (BMF) to enhance cell survival. BIM is phosphorylated and dissociated from the pro-survival proteins MCL and BCL-XL. While the forkhead box O3 (FOXO3) phosphorylation (a transcriptional activator of both BIM and PUMA) promotes the ubiquitination and degradation of BIM and ultimately result in the ERK1/2-dependent suppression of BIM and PUMA expression (Balmanno and Cook, 1999). ERK1/2 signaling also promotes the phosphorylation and proteasome-dependent turnover of Bcl-2 associated death (BAD) protein (Chipuk et al., 2010). Caetano et al.’s (2008) work employing SN56 cells have shown the interaction of ERK1/2 and cellular prion protein in neuroprotection and increased neuritogenesis. Uppington and Brown (2008) have shown pERK1/2 upregulation in cerebellar granular neurons (CGN), N2a and scrapie-infected cell line (SMB). This upregulation of pERK1/2 were directly related to prion proteins as PrP knockout cells were not resistant to prion toxicity. Furthermore the inhibition of pERK1/2 resulted in an increased cell death which suggests the protective effect of increased pERK1/2 levels (Uppington and Brown, 2008). The apoptotic effect of ERK1/2 has been demonstrated in SH-SY5Y cells previously (Gao et al., 2014).

## B-The JNK Pathway

JNKs were initially identified as the kinases that phosphorylated and eventually activated the transactivation domain of the transcription factor c-Jun in response to the exposure of cells to ultraviolet (UV) treatment (Coffey, 2014). There are three major well known JNK genes that encode upto 10 different isoforms of JNKs through alternative splicing sites. Several cells in mammalian body ubiquitously express JNK1 and JNK2 genes. While JNK3 genes are primarily expressed in the brain cells (Xie et al., 2004; Coffey, 2014). JNK3 has also been found in insulin secreting cells and help in protection from apoptosis (Abdelli et al., 2009). The JNK isoforms are specific in binding to the substrate as the JNK1 preferentially bind to the c-Jun in contrast to other JNK isoforms such as JNK2 and JNK3 (Coffey, 2014). A variety of cellular stresses activate the JNKs such as UV, ionization radiation, methylating agents, translation inhibitors, heat shock and ER stress produced by misfolded proteins. The requirement for the JNK activation is dual phosphorylation of the Thr-Pro-Tyr motif by the upstream kinases MKK4 (which also activates p38) and MKK7. However, due to the identification of alternative splice variant sites at MKK7, it is differentially regulated by upstream signals and thus making the JNK regulation more complex (Coffey, 2014). There is a close relationship between JNK signaling and the death receptors and their ligands, together they cause apoptosis. However, JNK can also cause cell death via directly targeting the components of the BCL2 family members. For example under normal physiological conditions the pro-apoptotic BH3-only protein BIM is proposed to be sequestered by binding to the dynein motor complex; while in cellular stress response the BIM phosphorylation via JNK takes place within the dynein light chain binding motif results in its release from the dynein motor complex and subsequently triggering apoptosis (Figure 2; Coffey, 2014). Additionally the JNKs also promotes the phosphorylation of the BCL2 family members at both Ser and Thr motifs to initiate cell cycle arrest signaling at the G2/M-phase and subsequently reducing the resistance of the cells to apoptotic stimulus (Coffey, 2014). Based on these facts the JNK is generally termed as apoptotic pathway. However, the apoptotic signaling via JNK pathway alone is not sufficient enough to trigger apoptosis always and JNK signaling can also favor the survival of cell (Nix et al., 2011). The phosphorylation of BCL2 members at Thr69, Ser70 and Ser87 residues sites can promote the dissociation of BCL2 from Beclin1 ultimately promoting autophagy and cell survival signaling (Yamamoto et al., 1999). JNK1 plays an important role in spinal hyperalgesia model where the inhibition of JNK1 in spinal astrocytes substantially reduce pain sensation (Gao et al., 2010). JNK1 signaling is also necessary for guidance of cortical interneurons within cerebral cortex (Myers et al., 2014). The maintenance of neuronal microtubule and control of microtubule-associated proteins (MAPs) are essential for axonal and dendritic pruning. The JNK1 takes an active part in this process (Chang et al., 2003). Ries et al. (2008) showed that JNK2 and JNK3 were essential for apoptosis in dopaminergic neurons. The JNK2 and JNK3 double null mutations resulted in abrogation of apoptosis and prolonged survival. But this protection was only limited to soma of the neurons and not to axons (Ries et al., 2008).

## C-The p38 Pathway

The p38 MAPKs activation has been observed in response to a variety of inflammatory cellular stresses such as hyperosmolarity, arsenite or taxol and pro-inflammatory cytokines released in response to activated microglia due to the accumulation of misfolded proteins (Zarubin and Han, 2005; Yang et al., 2015). The p38 MAPK pathway is activated after dual phosphorylation of a Thr-Gly-Tyr motif at Thr-180 and Tyr-182 residues and this reaction is catalyzed by MKK3, MKK4 and MKK6 but not the Ras/Raf/ Mitogen activated protein kinase/ERK kinase 1 and 2 (MEK1/2) signaling (Yamamoto et al., 1999). The p38 MAPK pathway consist of four isoforms in the adult mouse brain (α, β, γ, δ). These isoforms have been isolated from the brain tissues specifically cerebellum and cortex by using immunoblotting techniques (Correa and Eales, 2012). The p38α is found abundantly in neurons while p38β is found in glial cells. The p38α and p38β isoforms are ubiquitously expressed in many tissues of mammalian body including parts of the brain such as cerebral cortex and hippocampus. The p38γ and p38δ has a more limited tissue distribution and have different substrate preference as compared to the rest of the family members of p38 (Correa and Eales, 2012; Darling and Cook, 2014). The activation of p38 can either be cell proliferative or it can induce apoptosis via cell cycle arrest. All the signaling cascades of p38 depends on the kinetics of activation and downstream signaling pathways that are activated in response to specific stimuli (Kato et al., 2013). The growth factor induced transient stimulation of p38 pathway result in the promotion of cyclin D1 expression and phosphorylation of the transcriptional repressor proteins known as retinoblastoma associated protein (Rb; Balmanno and Cook, 1999). Upon phosphorylation, Rb is dissociated from the E2F motif and subsequent increased transcription of S-phase entry genes induce cell cycle proliferative changes (Qin et al., 1995; Sharma and Carew, 2004). In contrast, the prolonged activation of p38 which is seen in response to cellular stresses such as glumate-induced stress and misfolded proteins accumulation stress leads to the activation of MAPK-activated protein kinase-2 (MK-2) and its substrate CREB (Chaparro-Huerta et al., 2008). This promotes cell cycle arrest perhaps through the induction of CDK inhibitors p16, p21 or p27 (Darling and Cook, 2014). The p38 MAPK pathway in conjunction with the JNK pathway leads to cell cycle arrest and ultimate cell death through phosphorylation of the proapototic protein BIM (Sharma and Carew, 2004). The BIM protein expression is promoted through the activation of the transcription factor FOXO3a (Darling and Cook, 2014). Additionally, p38 promotes the phosphorylation of the transcriptional activator p53 and enhance its stabilization and transcriptional activity. The increased expression of p53-targeted genes promotes an increased apoptosis. The apoptosis occur under the effect of the pro-apoptotic proteins PUMA and Phorbol-12-myristate-13-acetate-induced protein 1 (PMAIP1) also known as NOXA (Figure 2; Bulavin et al., 1999).

## Role of The MAPK Pathways in Alzheimer’s and Parkinson’s Disease

Activation of the ER stress associated MAPK pathways has been reported in several neurodegenerative diseases including AD, PD, amyotrophic lateral sclerosis and prion diseases (Kim and Choi, 2015). AD is a slowly progressive disease of the brain that is characterized by symptoms like impairment of memory and eventually by disturbances in reasoning, planning, language, and perception. The Risk of developing AD increases substantially after the age of 70 and it may affect around 50% of persons over the age of 85 in United States. Over the past few years p38 MAPK pathway has been extensively studied to devise the treatment for AD (Munoz et al., 2007). This may be due to the possible involvement of p38 MAPK pathway in the context of neuroinflammation and subsequent cytokines release in response to the amyloid beta accumulation (Munoz and Ammit, 2010). Munoz et al. (2007) have shown that orally bio-available small molecules lead to the suppression of brain pro-inflammatory cytokines via p38 MAPK signaling which attenuated synaptic dysfunctions and behavioral deficits in AD mouse model. The relationship of p38 MAPK activation with early neurofibrillary degeneration in AD post mortem brain showed that early neurofibrillary tangles were associated with the activation of phosphorylated p38 but not the senile plaques (Sun et al., 2003). Bell et al. (2004) found that MAPK ERK activation is dependent on the duration and physical status of amyloid beta exposure in hippocampal slices. Furthermore low molecular weight short term exposure to amyloid beta did not activate MAPK JNK pathway. The large molecular weight long term exposures lead to the activation of MAPK JNK coincident with MAPK ERK down-regulation (Bell et al., 2004). Leugers et al. (2013) recently demonstrated that tau plays a major role in signal transduction processes after activation by NGF in PC12-derived cells. Depletion of tau resulted in the attenuation of NGF-induced MAPK activation and restoration of tau caused the activation of MAPK pathway. This activation of tau required successful phosphorylation of tau at Thr231 site (Leugers et al., 2013). Recent studies demonstrated that α7nAChR binds to soluble amyloid beta with a high affinity. *In vitro* and *in vivo* experiments have also shown that amyloid beta activates p38 MAPK and ERK1/2 signaling pathways via the a7nAChR. Furthermore, these findings suggest that a7nAChR and MAPK signaling pathways play an important role in the uptake and accumulation of amyloid beta1–42 in SH-SY5Y cells (Yang et al., 2014). Repeated intra-hippocampal injections of amyloid beta activated caspase-3 and MAPK signaling cascade to produce behavioral deficits (Ghasemi et al., 2014).

PD belongs to a group of diseases called motor system disorders caused by the loss of dopaminergic neurons in the brain. PD is a chronic neurodegenerative disorder with the second largest incidence rate among all other neurodegenerative disorders only after AD. Currently there is no effective therapy available for PD cure and several research groups are focusing to investigate the therapeutic prospect in cell cultures and animal models of PD. There are many factors contributing towards the pathogenesis of PD but the role of p38 MAPK and PI3K/AKT signaling cascade in PD brains has been crucial. This may be due to the fact that impaired balance between the pro-survival and pro-death pathways which trigger the activation of microglia and subsequently result in neuroinflammation, oxidative stress and loss of dopaminergic neurons (Jha et al., 2015). Previous studies employing N2a cell line showed the suppressive effect of α-synuclein on MAPK pathways. Furthermore this suppression led to neuronal apoptosis which was prevented successfully with the transfection of active mitogen activated protein kinase 1 (MEK-1; Iwata et al., 2001). Yshii et al. (2015) reported that SH-SY5Y cells transduced with α-synuclein (WT or A30P) and treated with conditioned medium (CM) from LPS-activated glial cells have shown the evidence of cell death that was abolished with the inhibition of the MEK1. The glial cells activation through the p38 MAPK-MK2 signaling cascade consequently result in the production of inflammatory cytokines such as tumor necrosis factor alpha (TNF-α). The TNF-α can induce and promote neuroinflammation as a physiological neuroprotective mechanism. If the glial cells become overactivated through elevated levels of extracellular α-synuclein released from damaged Lewy body containing neurons may then lead to chronic inflammation and neuronal cell death (Correa and Eales, 2012). PD model employing SH-SY5Y cells model infected with rotenone induced phosphorylation of the two main branches of MAPK pathways; the c-JNK, p38 MAPK and the c-Jun. This indicates the activation of the p38 and JNK pathways. Interestingly, rotenone-induced apoptosis was diminished by the expression of dominant interfering constructs of the JNK or p38 pathways. These data indicate the possible role of JNK and p38 MAP kinases and caspases in retonone-induced apoptotic signaling in the dopaminergic SH-SY5Y cells (Newhouse et al., 2004). The involvement of active phosphorylation-dependent mitogen-activated protein kinase (MAPK/ERK), stress-activated protein kinase/c-Jun N-terminal kinase (SAPK/JNK) and p38 kinase expression in PD and Dementia with Lewy bodies have been reported by Ferrer et al. (2001).

Apart from AD’s and PD’s, several research groups worked on the role of MAPK pathways in other neurodegenerative disease conditions, such as neuronal injury models (Arboleda et al., 2010; Jantas et al., 2011; Lin et al., 2011; Liu et al., 2014), spinal cord injury models (Li et al., 2014), neuronal toxicity studies (Mao et al., 2016), oxidative stress and inflammation models (Nafees et al., 2015), experimental diabetic neuropathy models (Zhou et al., 2016) and synaptic depression studies (Sharma and Carew, 2004; Lippiello et al., 2016). So the role played by the MAPK signaling cascade cannot be neglected in all major neurodegenerative diseases especially those with the history of misfolding in the ER.

## The Mechanisms of Cell Death and Role of The MAPK Pathways in Prion Diseases

TSEs are thought to be caused by a protein termed the prion protein. This protein was first discovered in 1982 by Prusiner and is still unique as it is the only protein thought to be a transmissible disease causing protein. The prion protein is a 30–39 kDa protein that is expressed in most tissues. The highest expression levels are found in the neural and lymphoid tissue. In prion diseases the cellular prion protein (PrP^c^) misfolds and transform to prion protein scrapie (PrP^Sc^). There is an increase in the β- sheet content and a corresponding decrease in α-helices (Song et al., 2016). The structural changes from PrP^c^ to PrP^Sc^ corresponds to the loss of function and perhaps most importantly for disease perspective the protease resistance of PrP^Sc^ and consequent aggregation leads to the stress stimuli within ER (Prusiner, 1998). The misfolded form of the PrP^c^ is thought to be the sole cause of neurodegeneration in the TSE’s although the underlying mechanisms needs further molecular research.

**Figure 3:**
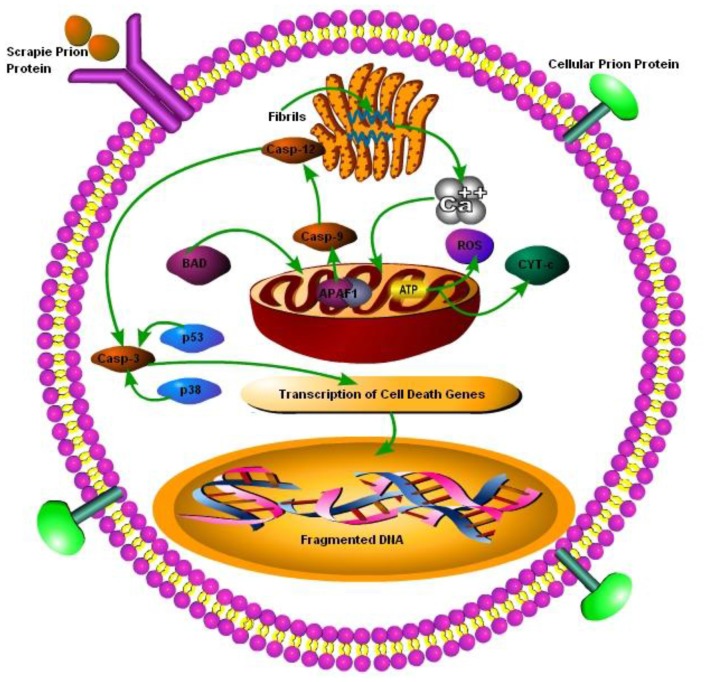
**Schematic presentation of cell death mechanisms in prion disease.** The cellular PrP (PrP^c^) is found on the cell membrane, upon change from PrP^c^ to prion protein scrapie (PrP^Sc^), it is internalized by cell membrane bound receptors and transported to ER for folding and processing. The accumulation of misfolded PrP^Sc^ in ER leads to perturbations in calcium (Ca^2+^) homeostasis, which ultimately leads to pro-apoptic BCL-2 associated death (BAD) translocation into mitochondria and release of cyctochrome-c and apoptotic activating factors from mitochondria, triggering caspase-dependent apoptotic signaling. Dysregulated Ca^2+^ homeostasis results in reduced ATP production in mitochondria leading to reactive oxygen species (ROS) production. Under caspase-dependent apoptotic surge caspe-3 is degraded via p53 and p38 transcription factors leading to DNA fragmentation in nucleus of the cell (Ferreiro et al., 2006, 2008a,b; Uppington and Brown, 2008).

Prion diseases are characterized by massive neuronal cell apoptosis leading to the vacuolation of brain and subsequent classical spongiform appearance in the brain tissues. However, despite intensive research the mechanisms of prion-protein-induced neurotoxicity are still unclear. The neurotoxicity in prion diseases may not be wholly the result of the loss of function of the prion protein as PrP null mice were found to be normal. Although as conversion from PrP^c^ to PrP^Sc^ is thought to result in the loss of function, only it may have some role in neurotoxicity as well (Soto and Satani, 2011). Previously Taraboulos et al. (1992) have shown that ER is not a component of the synthesis of PrP^Sc^ and the synthesis occurs during the transit of PrP^c^ between the mid-golgi stack and lysosomes. It is still unclear how the aggregation of misfolded PrP^Sc^ replication triggers ER stress. The possibility that abnormal prions directly target the ER remains to be explored. Recently it has been discovered that there is a strong link between ER stress due to the accumulation of misfolded proteins and subsequent UPR activation (Ferreiro et al., 2006, 2008b; Moreno et al., 2012, 2013; Hetz and Mollereau, 2014). The presence of PrP^c^ at the cell surface suggests that these proteins may have a role in the transduction of cell signaling from cell surface towards the inner side of cell. In lymphocytes, PrP^c^ has been shown to be incorporated into lipid rafts within the cell membrane and these rafts contain accumulated cross-linked proteins (Laurén et al., 2009). It is hypothesized that PrP^c^ may become cross-linked in these rafts. It has been have shown that scrapie infection triggers an abnormal subcellular distribution of PrP at the terminal stage of the disease and PrP^Sc^ is no more found at lipid rafts and is translocated to cytosol (Russelakis-Carneiro et al., 2004). Similarly Yang et al. (2006) have shown that yeast cytoplasm contain PrP^Sc^ and this PrP^Sc^ had self replicating property as it convert normal cellular PrP^c^ to proteinase-k resistant conformation. The PrP^c^ has been found to act as a receptor for Aβ in AD and synaptotixicity and cognitive impairments were also mediated by PrP^c^ (Gimbel et al., 2010). Ashok and Hegde (2008) have shown that retrotranslocation of ER resident prion proteins occurs by prevention of GPI signal transamidation. The PrP^c^ cross-linking has been shown to induce the activation of several signaling pathways including MAPK pathways (Puig et al., 2016). It is still unclear that PrP^Sc^ itself is toxic as demonstrated by Hetz et al. (2008), or it is not as toxic by itself but it activates neurotoxic signaling pathways (Soto, 2003). The Bcl-2 proteins family comprises both the anti-apoptotic and pro-apoptotic signaling molecules; the regulation of apoptosis occurs via the release of factors such as apoptotic activating factors (APAFs) and cytochrome-c from the mitochondria. Bcl-2 an anti-apoptotic signaling molecule and Bax a pro-apoptotic signaling molecule have been implicated in prion toxicity. Over-expression of the anti-apoptotic protein Bcl-2 in GT1 cells alleviated the neurotoxic effects of the 106-126 prion peptide (Ferreiro et al., 2007). Several studies shown the ER-mitochondria cross-talk in prion diseases and the role played by dysregulated calcium homeostasis in propagation of prion infection (Ferreiro et al., 2006, 2008a,b). Reactive oxygen species (ROS) production and loss of adenosine triphosphates results in the release of the cyctochrome-c from mitochondria. As a result BAX translocation into the mitochondria triggers apoptotic activation factors and subsequent caspase-9, caspase-12 and caspase-3 activation initiates apoptosis (Figure 3; Ferreiro et al., 2008a).

Previous reports showed the activation of MAPK pathways in response to misfolded prion protein accumulation in brain of the prion infected animals. The toxic prion peptide PrP106-126 resulted in up-regulation of pJNK (Carimalo et al., 2005). Also pJNK and pERK was found up-regulated in the brains of hamsters infected with 263k strain of scrapie (Lee et al., 2005). Several models also have shown that p38 MAPK is activated (Thellung et al., 2002; Lee et al., 2005). The activation of p38 may lead to caspase 3 activation and apoptosis as inhibition of p38 has been shown to prevent cell death in cells treated with the prion peptide PrP106-126 (Thellung et al., 2002). Uppington and Brown (2008) have shown that scrapie infected cells had elevated caspase-12 expression and an increased level of the phosphorylated ERK was neuroprotective as inhibition of pERK resulted in increased cell death. Furthermore pERK activation was solely dependent on membrane-resident PrP proteins (Uppington and Brown, 2008). CCR1 knocked-out mice experiment shown rapid prion disease progression in knocked-out mice as compared to wild type mice. Additionally CCR1 knocked-out mice had increased ERK1/2 activation. This shows the importance of ERK1/2 signaling in prion diseases pathogenesis (LaCasse et al., 2008). N2a and NT2 cell lines treated with nitric oxide shown p38 MAPK pathway activation and an increase in PrP^Sc^ level which was reduced when special p38 MAPK inhibitor was given (Wang et al., 2005). More recently Puig et al. (2016) have shown that the mutant prion protein was retained in the secretory pathway and induced the activation of p38-MAPK pathway and caused a lethal disease in mice model of prion diseases.

Altogether, these data suggest the link between misfolded prion protein accumulations and subsequent ER-mitochondria cross-talk in face of chronic ER stress and UPR activation. Furthermore, a strong link between UPR and MAPK pathways does exist as shown by previous *in vitro* and *in vivo* reports in prion diseases.

## Conclusions and Future Perspectives

There are many evidences suggesting the role of misfolded prion proteins in the initiation of ER stress mediated IRE1, PERK and ATF6 pathways. The adaptive response to ER stress is called UPR which is aimed at correcting overall protein processing in order to reduce the accumulation of misfolded proteins and restore the normal cellular functions. If ER stress is too severe or if the adaptive response fails to compensate the ER stress produced then synaptic dysfunction and apoptosis occurs. There are clearly strong links between the UPR signaling cascades and MAPK pathways in all major protein misfolding disease including prion diseases. In some cases the UPR signaling results in the activation of MAPK pathways so the UPR act as a trigger for the MAPKs; in addition, there are several other pathways where many interaction points allow cross talk between these pathways. Accumulation of misfolded proteins initiates the UPR and promote the activation of ERK1/2 under the effect of IRE1 pathway on one hand and JNK activation through IRE1-TNF receptor associated factor 2 (TRAF2)-ASK signaling or due to the Ca^2+^ dysregulation in response to ER stress. In addition the modulation of UPR can occur through the activation of MAPK p38 and subsequently phosphorylating CHOP and ATF6 proteins. Generally the ERK1/2 activation is considered to promote cell survival signaling. In contrast the JNK and p38 pathway signaling is considered pro-apoptotic. However, several exceptions do exist; for example, JNK signaling under the influence of IRE1 pathway promotes c-Jun activity and the expression of pro-survival protein Adapt78. Additionally ERK1/2 signaling cascade protect the cell against ER stress stimulus (such as in prion protein infected N2a and SN56 cell line and melanoma cells). But there are several cases where ERK1/2 can be apoptotic (such as colorectal melanoma cell line, HCT116 cell line, or the neuroblastoma cell line SH-SY5Y). The mechanism of MAPK signaling requires further molecular research to determine whether MAPK-dependent signaling can promote cell proliferation, survival or cell death. Scientists are agreed on the analogy with the other investigations that the response to ER stress could prove crucial in reflecting the differences in the duration of signaling, magnitude of signaling, relationship with other signaling pathways and to some extent cell type-specific expression of MAPK cascades. Although there are still several challenges in understanding the role of MAPK signaling and ER stress in prion diseases there are cases where there are indications that therapeutics to manipulate both UPR and MAPK signaling could be advantageous. To date studies on prion disease *in vivo* models or *in vitro* models none has focused on inhibiting UPR and MAPK pathways simultaneously. Moreno et al.’s (2013) work on mice model of prion disease focused on the role of PERK pathway or Orsi et al.’s work on IRE1α pathway have clearly shown the importance of PERK and IRE1-XBP1 pathways in prion misfolding diseases. They have shown that PERK inhibition and XBP1 overexpression resulted in the alleviation of ER stress (Orsi et al., 2006; Moreno et al., 2012, 2013). On the other hand Thellung et al.’s work on an *in vitro* model of prion diseases has shown the prosurvival effects of p38 MAPK inhibition in SH-SY5Y cells. This neuroprotective effect of SB203580 and PD169316 (p38 Inhibitors) reversed caspase mediated cell death in prion disease cell model (Thellung et al., 2002). Similarly pJNK has been found to be upregulated both in *in vivo* and *in vitro* model of prion diseases (Carimalo et al., 2005; Lee et al., 2005). ERK1/2 phosphorylation is yet to be understood in prion diseases as Lee et al. (2005) and Uppington and Brown (2008) shown pERK upregulation in prion diseases, and this upregulation was neuroprotective.

It is clear from previous investigations that useful therapeutic investigation for all the major misfolding diseases such as AD, PD, HD and TSEs should be focused on targeting either the misfolded proteins directly or indirectly by targeting the subsequent downstream signaling pathways. These pathways are initiated in response to the ER stress due to accumulation of the pathogenic proteins. UPR and MAPK pathways need further molecular research to exactly pinpoint the signaling cascades that are activated in response to the accumulation of prion protein in TSEs. In addition, focusing on the UPR and MAPK pathways simultaneously might be a good idea as all the previous research conducted on prion diseases was focused solely either on UPR inhibition or MAPK inhibition individually. Modern molecular level pharmacological research has contributed significantly towards achieving the goal of inhibiting MAPK pathways. Currently all available inhibitors in the market can only target any one of the arms of MAPK pathways. By contrast to other arms of MAPK pathways, many p38 MAPK inhibitors are available such as PH-797804, BIRB 796, VX-702, SB 239063, SB202190, SCIO 469, and BMS 582949. ERK1/2 pathway inhibitors available are PD98059, BVD-523 and U0126. JNK pathway inhibitor commonly used in research is SP600125. On the other hand there are very few inhibitors available to target the UPR signaling cascades. That means simultaneous targeting of UPR and MAPK pathways via inhibitors may be difficult. But Modern scientific tools such as gene manipulation techniques (siRNA, miRNA) can be adopted to target UPR and MAPK pathways simultaneously in protein misfolding neurodegenerative diseases to achieve the goal of stress free environment within the nervous cell.

## Author Contributions

SZAS wrote the manuscript, DZ gave the idea, TH helped in figures and LY critically reviewed the manuscript before final submission.

## Conflict of Interest Statement

The authors declare that the research was conducted in the absence of any commercial or financial relationships that could be construed as a potential conflict of interest.
